# A Web Server and Mobile App for Computing Hemolytic Potency of Peptides

**DOI:** 10.1038/srep22843

**Published:** 2016-03-08

**Authors:** Kumardeep Chaudhary, Ritesh Kumar, Sandeep Singh, Abhishek Tuknait, Ankur Gautam, Deepika Mathur, Priya Anand, Grish C. Varshney, Gajendra P. S. Raghava

**Affiliations:** 1 Bioinformatics Centre, CSIR-Institute of Microbial Technology, Sector 39A, Chandigarh, India; 2 CSIR-Central Scientific Instruments Organisation, Sector 30C, Chandigarh, India; 3 Cell Biology and Immunology Division, CSIR-Institute of Microbial Technology, Chandigarh, India

## Abstract

Numerous therapeutic peptides do not enter the clinical trials just because of their high hemolytic activity. Recently, we developed a database, Hemolytik, for maintaining experimentally validated hemolytic and non-hemolytic peptides. The present study describes a web server and mobile app developed for predicting, and screening of peptides having hemolytic potency. Firstly, we generated a dataset HemoPI-1 that contains 552 hemolytic peptides extracted from Hemolytik database and 552 random non-hemolytic peptides (from Swiss-Prot). The sequence analysis of these peptides revealed that certain residues (e.g., L, K, F, W) and motifs (e.g., “FKK”, “LKL”, “KKLL”, “KWK”, “VLK”, “CYCR”, “CRR”, “RFC”, “RRR”, “LKKL”) are more abundant in hemolytic peptides. Therefore, we developed models for discriminating hemolytic and non-hemolytic peptides using various machine learning techniques and achieved more than 95% accuracy. We also developed models for discriminating peptides having high and low hemolytic potential on different datasets called HemoPI-2 and HemoPI-3. In order to serve the scientific community, we developed a web server, mobile app and JAVA-based standalone software (http://crdd.osdd.net/raghava/hemopi/).

Therapeutic peptides have a number of advantages over traditional drugs that are mainly based on small molecules and antibodies^1^. In the recent years, a large number of databases have been published, revealing discovery of novel peptides with therapeutic properties ranging from antimicrobial^2^,^3^,^4^, antimalarial^5^, antiparasitic^6^, anticancer^7^, cell penetrating^8^, tumor homing^9^, antihypertensive^10^ etc. Peptides have gained leverage over antibody and small molecule-based drugs as they possess better tissue penetration, high specificity and comparatively low production cost^11^,^12^. Though hundreds of potential therapeutic peptides have been discovered so far, only limited peptide-based drugs are in the market.

The major hurdles preventing therapeutic peptides from translating into drugs are their high toxicity, low metabolic stability and poor oral bioavailability^1^. In order to improve the stability of a peptide, a method HLP has been developed for designing peptides of desired half-life^13^. Toxicity is one of the major hurdles in designing peptide-based therapeutics^14^. The toxicity of peptides can be broadly classified into three categories, namely cytotoxicity, hemotoxicity (lysis of red blood cells) and immunotoxicity. Recently, a method, ToxinPred, has been developed for predicting toxins (toxic proteins/peptides) and identification of residues/segments responsible for toxicity^15^. In the past, numerous methods have been developed for predicting different types of epitopes that can be used for predicting immunotoxicity/allergenicity of peptides^16^,^17^,^18^,^19^. To the best of our knowledge, limited attempts have been made so far to develop methods predicting hemolytic potency of peptides^20^,^21^,^22^,^23^. Experimental approaches for determining hemolytic potency of a large number of peptides is a labour-intensive, time-consuming and costly affair. *In silico* models may provide an alternative to the tedious experimental approach for predicting hemotoxicity of peptides. Recently, our group collected and compiled hemolytic peptides from the literature and various other resources, in order to create a repository of experimentally validated hemolytic peptides^24^. In the present study, we developed various models for predicting hemotoxicity of peptides using different machines learning techniques. These models have been developed using various peptide features that include residue-based compositions, binary profiles, and hemolytic motifs. Further, we developed *in silico* tools for researchers working in the field of therapeutic peptides.

## Methods

### Datasets

For generating datasets, experimentally validated hemolytic peptides were extracted from Hemolytik database^24^ which is a comprehensive collection of peptides with their hemolytic potencies compiled from individual labs around the world using different protocols. Firstly, peptides were extracted having only natural amino acids. Since Hemolytik database covers peptide entries showing varing hemolytic potencies, peptides fulfilling the following criteria were considered as highly hemolytic and rest were considered as non-hemolytic.

Criteria for considering highly hemolytic peptides:

1. Half maximum Effective Concentration (EC_50_) or Hazardous Concentration (HC_50_) ≤100 μM
2. Minimum Hemolytic Concentration (MHC) ≤ 250 μg/ml
3. >10% hemolytic activity upto 100 μM

#### HemoPI-1 dataset

It consists of 552 experimentally validated hemolytic peptides, extracted using above criteria from Hemolytik database as positive examples. For negative examples, an equal numbers of peptides were randomly extracted from proteins in Swiss-Prot^25^ while ensuring similar length distribution as that of positive examples. This dataset was randomly divided into 2 sub-datasets named ‘HemoPI-1 main’ dataset (80% data with 442 positive and equal numbers of negative examples) and ‘HemoPI-1 validation’ dataset (20% data with 110 positive and equal numbers of negative examples).

In order to make sure that random negative data is not biased, we created additional 5 random negative datasets (Random1, Random2, Random3, Random4, and Random5). These datasets were of equal size as that of HemoPI-1 negative dataset. Random negative datasets 1–5 were created using same procedure as that of HemoPI-1 negative dataset.

#### HemoPI-2 dataset

To develop a method for discriminating peptides exhibiting high and low hemolytic potency, a second dataset named ‘HemoPI-2’ was created. In this dataset, instead of extracting peptides randomly from Swiss-Prot, a total of 462 peptides, which exhibited low hemolytic potency or peptides which do not fulfil the above criteria, were extracted from Hemolytik database. This dataset was also divided into 2 sub-datasets named ‘HemoPI-2 main’ dataset (80% data with 442 positive examples and 370 negative examples) and ‘HemoPI-2 validation’ dataset (20% data with 110 positive examples and 92 negative examples).

#### HemoPI-3 Dataset

In addition to the above two datasets, we have created third dataset HemoPI-3, which consists of 1623 peptides. In order to create HemoPI-3, we extracted 686 unique peptides from database DBAASP v.2 (Database of Antimicrobial Activity and Structure of Peptides)^26^ and 937 unique peptides from database Hemolytik. We defined highly hemolytic and poorly hemolytic peptides using criteria given in Supplementary Table S1. Based on this criteria, we got 885 highly hemolytic (taken as positive examples) and 738 poorly hemolytic peptides (taken as negative examples). This dataset was randomly divided into 2 sub-datasets (ensuring similar length distribution) named ‘HemoPI-3 main’ dataset (80% data with 708 positive and 590 negative examples) and ‘HemoPI-3 validation’ dataset (20% data with 177 positive and 148 negative examples).

### Cross-validation technique

The validation is an essential part of any prediction method. In the present study, a five-fold cross-validation technique was used to evaluate the performance of all the models. Here, sequences are randomly divided into five sets, of which four sets are used for training and the remaining fifth set for testing. The process is repeated five times in such a way that each set is used once for testing.

### Machine learning approaches

We have used machine learning approaches like SVM^*light*^ (Support Vector Machine)^27^, IBK (Nearest Neighbor)^28^, Multilayer Perceptron^29^, Logistic^30^, J48^31^ and Random Forest^32^ for the prediction of hemolytic peptides. SVM^*light*^ is a freely available program based on support vector machine and allows the user to perform classification with different kernels (polynomial, radial basis function, sigmoidal function) and parameters. IBK, Multilayer Perceptron, Logistic, J48 and Random Forest algorithms were implemented using WEKA package^29^.

### Input features for prediction

Any classification algorithm requires a set of fixed length of input features for training, thus necessitating a strategy for encapsulating the global information about peptides of variable length in a fixed length format. The fixed length format was obtained from peptide sequences of variable length using amino acid composition (vector size 20), dipeptide composition (vector size 400) and binary profile of pattern (vector size 20 for each residue). The formulae to calculate these features are described elsewhere^19^,^33^.

### Two sample logos

The two sample logos were generated using online two sample logo software^34^. These logos provide the position specific frequency of amino acids in a peptide. Each logo consists of stacks of symbols, one stack for each position in the sequence. The overall height of the stack indicates the sequence conservation at that position while the height of symbols within the stack indicates the relative frequency of each amino acid at that position.

### Quantitative Matrix

Positional preferences of residues have been used and represented earlier in the literature in the form of quantitative matrices (QMs)^15^. QMs show the propensity of each amino acid/dipeptide/property at each position in the positive (hemolytic) and negative (non-hemolytic) datasets. Three types of QMs have been generated:

#### Single residue-based

For each position, fraction of each amino acid is calculated for both positive and negative datasets. The final propensity value in each cell represents the subtraction of fraction of a particular residue in negative dataset from the fraction of that residue in the positive dataset. Thus a resultant matrix is of dimension N × M is formed, where N and M represent rows (number of single residues) and columns (number of positions; first 30 positions are taken into account) respectively. So in HemoPI, at single amino acid level, QM has dimensions of 20 × 30.

#### Dipeptide-based

Here, for each position, fraction of each possible dipeptide is calculated for both positive and negative datasets and final propensity value represents the subtraction of fraction of a particular dipeptide in negative dataset from the fraction of that dipeptide in the positive dataset. Thus a resultant matrix is of dimensions 20 × 29.

#### Property-based

Here, for each position, fraction of residues falling into a particular category of physicochemical properties (11 categories in total) is calculated for both positive and negative datasets. The final propensity value represents the subtraction of fraction of those residues in a particular class in negative dataset from the fraction of residues in positive dataset. Thus the resultant matrix is of dimensions 11 × 30.

Above three types of matrices have been calculated for both HemoPI-1 and HemoPI-2 datasets.

### Hybrid approach

For better and biologically reliable prediction, we integrated motif-based approach with the machine learning-based method. We used “Motif—EmeRging and with Classes—Identification” (MERCI) software^35^ to extract motifs exclusively present in hemolytic and non-hemolytic peptides. In the case of HemoPI-1 dataset, we extracted motifs exclusive to hemolytic peptides as the negative dataset of HemoPI-1 comprises of random peptides from Swiss-Prot while in case of HemoPI-2 dataset, motifs exclusive to hemolytic as well as non-hemolytic peptides were extracted. The motifs were extracted only from main datasets and not from validation datasets. In order to obtain relevant motifs, we used only those motifs which were present at least in ten seqeunces in the dataset. To predict the test sequence as hemolytic or non-hemolytic, we added +1 or −1 in the SVM score if the motif is present in the test sequence from the hemolytic or non-hemolytic peptides respectively.

### Performance measures

A five-fold cross-validation technique was used to classify hemolytic peptides on main datasets. For performance evaluation on validation datasets, the model was developed on main dataset and applied on validation dataset. Standard performance parameters like Sensitivity (Sn), Specificity (Sp), Accuracy (Acc) and Matthews correlation coefficient (MCC), were used to evaluate the performance of the method. The formula to calculate these parameters are described elsewhere^33^.

### Physicochemical properties

The amphiphilicity and other physicochemical properties have been calculated using AAindex database^36^. Physicochemical properties (along with AAindex code/identifier) i.e. Hydrophobicity (EISD840101), Hydrophilicity (HOPT810101), Steric Hinderance (CHAM810101), Net Hydrogen (FAUJ880109), Solvation (EISD860101), Charge (KLEP840101), Hydropathy (KYTJ820101), Amphiphilicity (MITS020101) and Weight (FASG760101) were provided for display along with prediction output. For calculation of amphiphiliicty, hydrophilicity, hydrophobicity, steric hinderance, solvation and hydropathy properties, we took the average of the AAindex values corresponding to all residues in a given peptide. For instance, equation 1 describes the calculation of amphiphilicity:


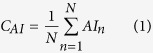


where, *C*_*AI*_ is the overall amphiphilicity of a peptide, *N* is the length of the peptide, *AI* is amino acid index value for amphiphilicity for *n*^*th*^ residue in that peptide.

For Weight, Net Hydrogen and Charge, we simply took the sum of AAindex values of all residues in a peptide.

## Results

### Amino acid composition analysis

**Figure 1:**
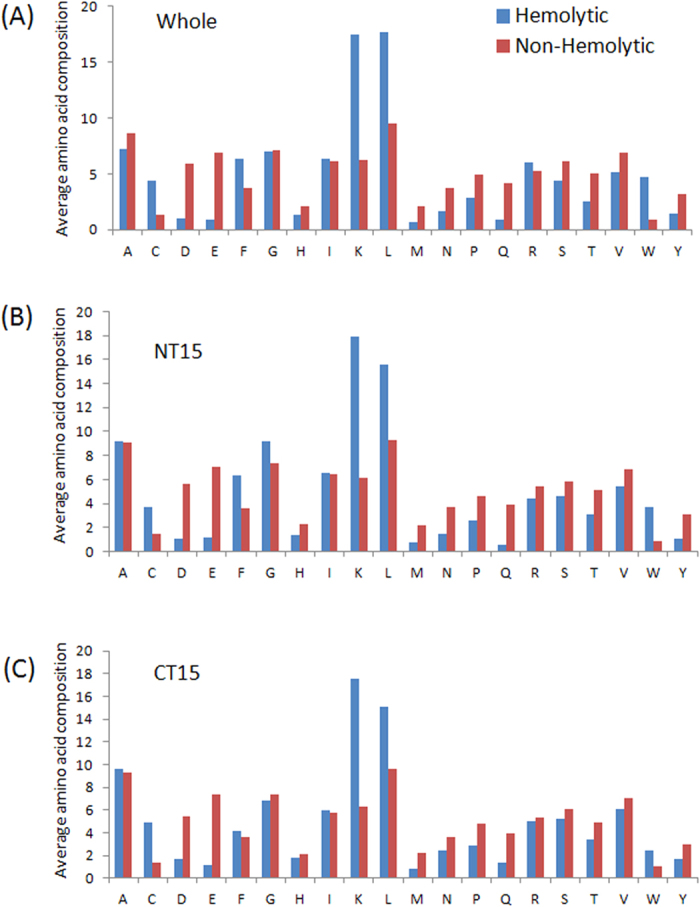
Amino acid composition of HemoPI-1 dataset peptides. Percent average amino acid composition of: (**A**) whole peptides, (**B**) N-terminal residues and (**C**) C-terminal residues between hemolytic and non-hemolytic peptides of HemoPI-1 dataset.

**Figure 2:**
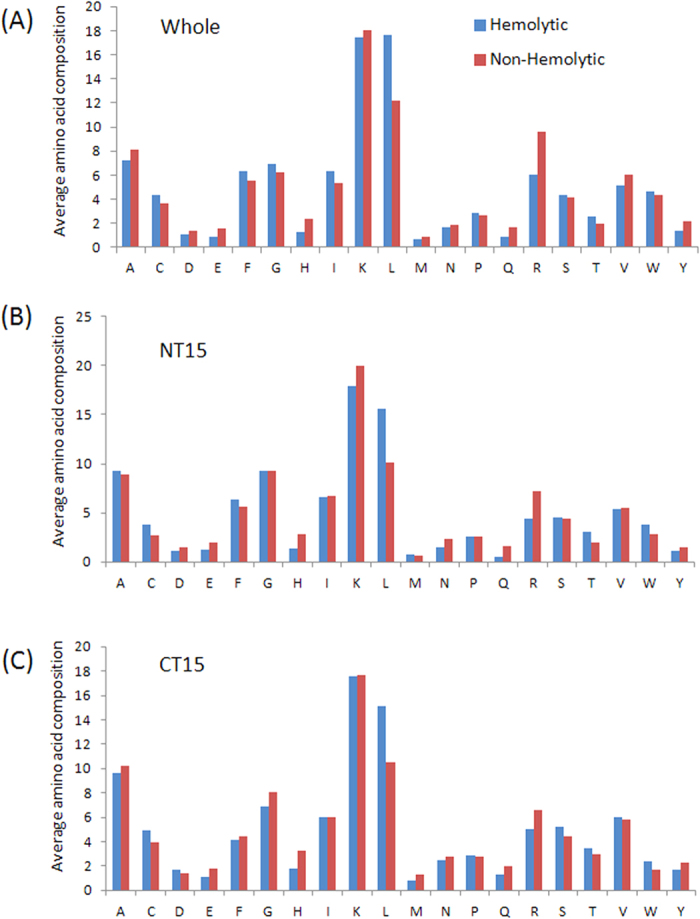
Amino acid composition of HemoPI-2 dataset peptides. Percent average amino acid composition of: (**A**) whole peptides, (**B**) N-terminal residues, and (**C**) C-terminal residues between hemolytic and non-hemolytic peptides of HemoPI-2 dataset.

First, to ascertain whether certain residues are dominated in hemolytic peptides, the percent average amino acid composition of hemolytic and non-hemolytic peptides were computed and compared. The results are shown in Figs 1 and 2. As seen in Figs 1 and 2, there are remarkable differences in the amino acid composition between hemolytic and non-hemolytic peptides. Certain residues like C, F, K, L and W were found to be dominating in hemolytic peptides as compared to non-hemolytic peptides in HemoPI-1 dataset (Fig. 1A). Percent average amino acid compositions at both the termini (spilt amino acid composition) were also compared and found to be similar to the whole composition (Fig. 1B,C) in HemoPI-1 dataset. In case of HemoPI-2 dataset, L was found to be more abundant in hemolytic peptides while R was found to be higher in proportion in non-hemolytic peptides (Fig. 2).

### Residue preference

**Figure 3:**
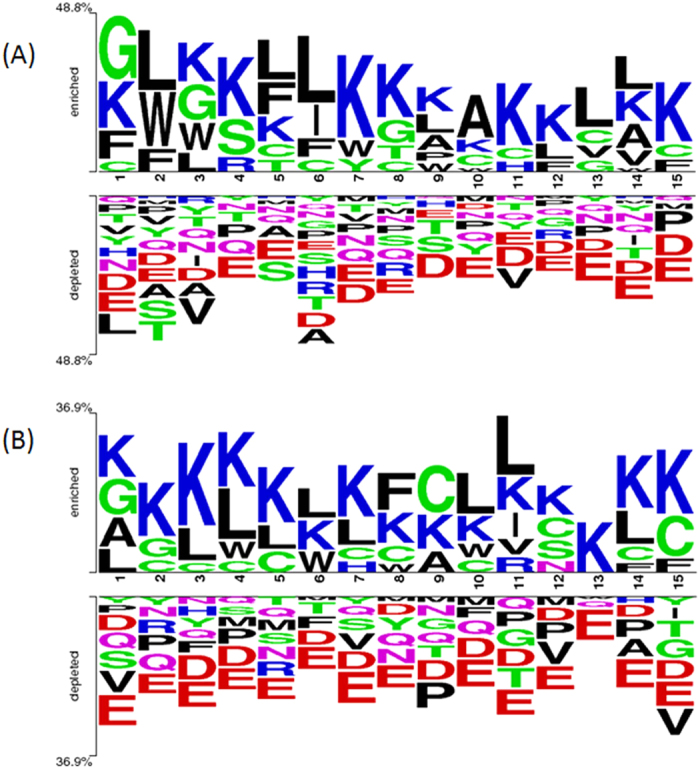
Two sample logos of hemolytic and non-hemolytic peptides of HemoPI-1 dataset. The figure depicts the two sample logos of: (**A**) first fifteen residues (N-terminus) and (**B**) last fifteen residues (C-terminus) of hemolytic (enriched) and non-hemolytic (depleted) peptides, where size of residue is proportional to its propensity.

**Figure 4:**
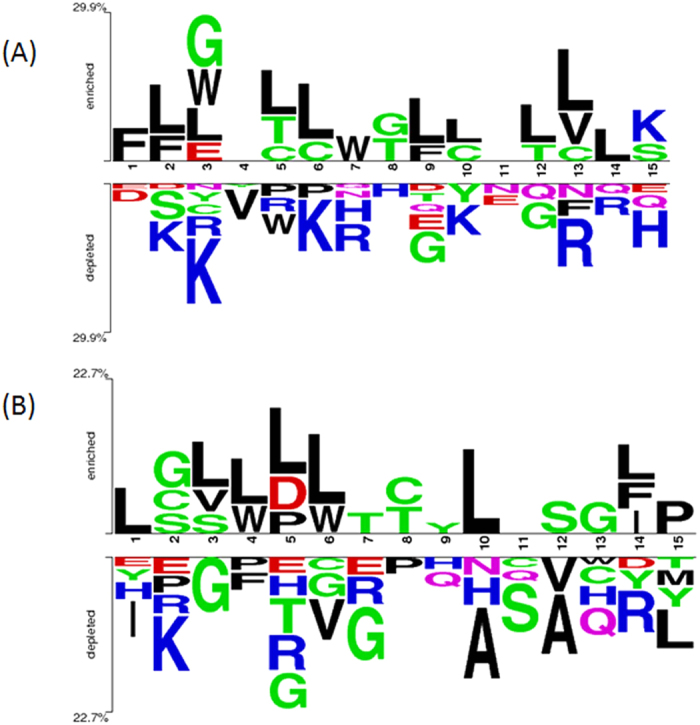
Two sample logos of hemolytic and non-hemolytic peptides of HemoPI-2 dataset. The figure depicts the sequence logos of: (**A**) first fifteen residues (N-terminus) and (**B**) last fifteen residues (C-terminus) of hemolytic (enriched) and non-hemolytic (depleted) peptides, where size of residue is proportional to its propensity.

We next wanted to understand the position-wise preference of residues at both the termini. Therefore, frequency of occurrence of all amino acids at both the termini was examined. We observed that certain types of residues are preferred over others in hemolytic and non-hemolytic peptides at N- and C-terminus. In order to demonstrate the residue preference at different positions of these peptides, two sample logos were generated. The two sample logos of 15 N-terminal and C-terminal residues of hemolytic and non-hemolytic peptides are shown in Figs 3 and 4 respectively. As seen in Fig. 3A, in case of HemoPI-1 dataset, a clear preference of certain residues at particular positions at N-terminus was observed in hemolytic peptides. For example, G, L and K are preferred at first, second and third positions respectively (Fig. 3A). However at other positions, L and K are preferred. Similar preference was observed at C-terminus (Fig. 3B). L and K are preferred at most of the positions. However, F and C were preferred at eighth and ninth positions respectively (Fig. 3B).

In case of HemoPI-2 dataset, at N-terminus of hemolytic peptides, a clear preference of hydrophobic residues was seen at various positions like F, L, and W are the most preferred residues at first, second and seventh positions while L is found to be preferred at most of the other positions (fifth, sixth, ninth, tenth, twelth, thirteenth and fourteenth positions) (Fig. 4A). On the contrary, at N-terminus of non-hemolytic peptides of HemoPI-2 dataset, overall hydrophilic residues are preferred at various positions like K is preferred at second, third, sixth, and tenth positions, R at seventh, thirteenth and fourteenth positions, and H at eighth and fifteenth positions. Only two positions (fourth and fifth) are found to be preferred by hydrophobic residues i.e. V and W. At C-terminus of hemolytic peptides of HemoPI-2 dataset, similar to N-terminus, L is preferred at most of the positions (first, third, fourth, fifth, sixth, tenth and fourteenth positions) while it was only preferred at one position (fifteenth) in non-hemolytic peptides (Fig. 4B). Rest of the positions were preferred by G (at third, fifth and seventh positions), A (at tenth and twelfth positions), I (at first), K (at second), F (at fourth) and V (at sixth positions) (Fig. 4B).

### Quantitative Matrix

#### Single residue-based QM

For HemoPI-1 dataset, at first position, residues F, G and K are preferred in positive dataset as compared to negative dataset. For the first 15 positions, residues K and L are preferred. In HemoPI-2 dataset, residue L is preferred at 2^nd^, 9^th^ and 13^th^ positions.

#### Dipeptide-based QM

At first position, FL dipeptide is preferred in HemoPI-1 dataset followed by KW. Similarly, FL is most preferred at first position (followed by IL) in HemoPI-2 dataset.

#### Property-based QM

First position is occupied by positively charged, large and hydrophobic residues in positive dataset, while negatively charged, small and polar residues are abundant in negative dataset in HemoPI-1 dataset. Similar to HemoPI-1 dataset, in HemoPI-2 dataset also, hydrophobic residues span positions from 1 to 17.

### Motif analysis

In order to understand whether any specific motifs are present in hemolytic or non-hemolytic peptides, we have extracted motifs using MERCI software. Sequence analysis of peptides in HemoPI-1 main dataset revealed the presence of “FKK”, “LKL”, “KKLL”, “KWK”, “VLK”, “CYCR”, “CRR”, “RFC”, “RRR”, “LKKL” etc., motifs specifically found in many hemolytic peptides. For extracting motifs which can discriminate between hemolytic peptides with high and low potency, we analysed sequence motifs in HemoPI-2 main dataset. Motifs like “ALW”, “GCS”, “AAAK”, “KLLS”, “LGKL”, “LLKKV”, “AKAAL”, “KVLKA”, “HIF” etc., are exclusively present in hemolytic peptides having high potency while motifs “KKG” and “QN” are present only in low potent hemolytic peptides. These motifs can therefore be used in the prediction algorithm to enhance the accuracy and reliability of the prediction of hemolytic/non-hemolytic peptides.

### Machine learning-based prediction models

The preliminary analysis suggested that hemolytic peptides can be discriminated from non-hemolytic peptides on the basis of amino acid composition, binary profiles and motifs. Therefore, various machine learning approaches were used to develop models using these features as input:

#### Amino acid composition-based model

In the past, amino acid composition as a feature has been used in various machine learning approaches to classify different types of peptides^15^,^16^,^19^. Therefore, first SVM models were developed using whole amino acid composition as input feature on HemoPI-1 and HemoPI-2 main datasets. The performances of these models are summarized in Table 1. For HemoPI-1, SVM model achieved an accuracy of 95.3% with MCC value 0.91. In case of HemoPI-2, SVM model achieved accuracy of 76.4% with MCC value 0.53 (Table 1). In case of HemoPI-3, we achieved an accuracy of 77.98% with MCC value 0.56 using SVM models (Supplementary Table S2). We also developed models using other classifiers like IBK, Multilayer Perceptron, Logistic, J48 and Random Forest using whole composition as input feature and performances of these models were found to be similar to the SVM-based model (Table 1 and Supplementray Table S3). Therefore, on rest of the features, models were developed using SVM only.

Next, we have created terminus-datasets (NT10, CT10, NTCT10, NT15, CT15 and NTCT15) consisting of first and last 10 and 15 residues from N- and C-terminus respectively as described elsewhere^33^. SVM models developed on these sub-datasets using amino acid composition as input performed more or less similar to the whole composition-based model (Table 2). For HemoPI-1, model achieved a maximum accuracy of 96.3% with MCC value 0.93 on NTCT15 dataset while in case of HemoPI-2, maximum accuracy of 77.5% with MCC value 0.55 on NTCT10 dataset (Table 2) was achieved. Similarly, on dataset HemoPI-3, our model achieved an accuracy of 81.0% with MCC value 0.61 on NTCT15 sub dataset (Supplementary Table S2).

In order to ensure that the random negative selection in HemoPI-1 dataset is not biased, we made 5 more random datasets and observed their average amino acid composition and prediction performance. The results were in agreement with that of the negative dataset taken in original HemoPI-1 negative dataset (Figure S1 and Supplementary Table S4). These datasets contain exact positive peptides but equal number of random negative peptides from Swiss-Prot. Average amino acid compositions for five random negative datasets (Random1-Random5) showed the similar trend as that of HemoPI-1 dataset (Supplementary Figure S1). Maximum accuracy ranged from ~93% to ~95% on main datasets (Supplementary Table S4).

#### Dipeptide-based model

Dipeptide composition encapsulates the global information of the amino acid fraction as well as the local order of amino acids and is used as feature in many prediction methods^15^,^19^. Thus, we have developed SVM models based on dipeptide composition on HemoPI-1 and HemoPI-2 main datasets which performed more or less similar to the composition-based models. Results are shown in Table 3. HemoPI-1 model achieved a maximum accuracy of 95.0% with MCC value of 0.90. For HemoPI-2, maximum accuracy achieved was 79.0% with MCC value of 0.58. SVM model achieved an accuracy of 82.38% with MCC value 0.64 on dataset HemoPI-3 (Supplementary Table S5).

#### Binary profile-based model

In preliminary analysis, it was found that certain residues are also preferred at various positions at N- and C-terminus. To incorporate this position specific information in the model, we have generated binary profile patterns of peptides. In binary pattern, a vector of dimension 20 represents a residue, and for n residues the input vector of dimension is 20 × n. Results are shown in Table 4. In case of HemoPI-1 main dataset, the binary model achieved a maximum accuracy of 95.7% with MCC value of 0.91. On HemoPI-2 main dataset, maximum accuracy achieved was 77.1% with MCC value of 0.54. SVM model achieved an accuracy of 80.57% with MCC value 0.60 on dataset HemoPI-3 (Supplementary Table S6).

#### Hybrid model

In order to improve the performance of the SVM hybrid model, we integrated the information obtained from motif analysis of HemoPI-1 and HemoPI-2 main datasets in the prediction of hemolytic and non-hemolytic peptides. The hybrid approach has been used in previous prediction methods to improve the prediction accuracy^15^,^37^. If there was no motif detected in the test sequence then amino acid composition was used as input feature for prediction using machine learning method. The results of hybrid approach are presented in Table 5. In the case of HemoPI-1, we achieved a maximum accuracy of 95.3% with MCC value of 0.91. For HemoPI-2, maximum accuracy achieved was 78.0% with MCC value of 0.56 (Table 5).

### Performance on validation dataset

In order to evaluate the performance of our best method we have created a validation dataset of 110 hemolytic peptides and equal number of non-hemolytic peptides, which have not been included in the training, feature selection and parameters optimization of the model. Our hybrid models achieved accuracy of 96.4% and 75.7% with MCC values 0.93 and 0.51 for HemoPI-1 and HemoPI-2 validation datasets respectively, as shown in Table 5, demonstrating that these models are useful or effective in real life.

## Implementation of Webserver

To the best of authors’ knowledge, no web service is available to date, which can predict hemolytic nature of peptides. Thus, in order to assist the scientific community, we have implemented our best method in a user-friendly web server ‘HemoPI’ with many other useful tools for the users. HemoPI has the following modules:

**(i) Hemolytic Potency:** This module predicts whether a given peptide is hemolytic or not. We have provided 5 options for the prediction. Model 2 (based on HemoPI-2 dataset, option 3) has been selected by default. User can select any of the 5 options to predict the haemolytic nature of the peptide. User has to submit the peptide sequence in single letter code and server will predict the hemolytic nature of the peptide. Besides the prediction, server will also generate all the possible substitution mutants (analogs) of query peptide with a single mutation in each mutant (depicted in red color) to find out the best analog with desired hemolytic acitivity. Server will rank the peptide analogs based on simple normalized score derived from the SVM score. SVM score has been transformed to obtain the normalized score which is directly proportional to the probability of a peptide being hemolytic. Peptides are ranked by probability, between 0 and 1, of being hemolytic i.e. 1 very likely to be hemolytic, 0 very unlikely to be hemolytic. This is like structure activity relationship which is often carried out in the wet lab to understand the crucial residues responsible for the activity. Therefore, this module is very helpful for the user in optimizing the hemolytic potency of therapeutic peptides. Along with this, server also calculates important physicochemical properties in an aesthetic tabular format.

**(ii) Virtual Screening:** This module works in a similar fashion to ‘Hemolytic Potency’ module. The only difference is that, in this module, user can submit multiple peptide sequences (in FASTA format) at a time. Thus, user can screen a library of peptides instead of a single peptide.

**(iii) Protein Mapping:** This is a very important module and useful to identify the various regions in a protein molecule, which can be hemolytic. For this, user may submit a protein sequence and server will first generate all possible overlapping peptide segments and then rank these peptides based on their scores. Thus, user can identify the possible hemolytic regions in a protein sequence.

**(iv) Quantitative Matrices:** ‘Q. Matrices’ module contains three types of matrices a) Single residue-based QM, b) Dipeptide-based QM and c) Property-based QM. These have been displayed for both HemoPI-1 and HemoPI-2 datasets. User can sort the specific positions to get an idea of most/least-preferred residues at each position for all three types of QMs. Numerical value in each cell is direct indicative of propensity in positive dataset. Numerical value-based gradient color-coding helps to easily identify the much-preferred residues (higher values in green color) and least preferred (negative values in chocolate color).

HemoPI web service is freely accessible at http://crdd.osdd.net/raghava/hemopi/.

### HemoPI Standalone Software

We developed JAVA-based standalone software in order to let the users apply and analyze the peptide sequences offline. The software has been developed on JDK 8 and has been tested to work on JDK 7 as well. It mainly consists of a back-end prediction engine and a front-end user interface. The prediction engine itself can be used as a standalone library and plugged in to achieve the classification results. Common to all the modules, users can either paste or write flat text file consisting of peptide sequences or upload them from the file system. The model file has to be uploaded which may be a LIBSVM or SVM^*light*^ trained model with different versions. The users can either explicitly state the model file version and type or leave it for automatic detection if not known beforehand. The results will either be stored in a user supplied result file or a result file automatically. The users can select the physicochemical properties to be calculated for the results. The results are displayed in a table where each row consists of peptide sequences, their probability scores along with the different physicochemical properties selected by the user. Each row when clicked creates a recursive list of all the possible combination of valid peptide sequences with the same columns as in the parent table. The values in the columns can be sorted by clicking the column tabs. We have also provided an example to show the sample result table and process.

The software has been bundled as zipped jar files having all the necessary libraries and example files, which can be downloaded from http://crdd.osdd.net/raghava/hemopi/HemoPI.zip.

### HemoPI Mobile App

We have also developed mobile application ‘HemoPI’ which can be installed on the android mobile phones. This app contains two modules that are described below:

**(i) Predict:** This module is used to predict the hemolytic property of the input peptide sequence. A user needs to type the peptide sequence followed by pressing/tapping on the ‘Predict’ button. In the next step, the result is displayed as the probability score of the query peptide.

**(ii) Mutant:** This module is used to generate mutant analogs of the input peptide sequence and predict the hemolytic nature of each of them. In this module, a user enters the peptide sequence, and presses/taps the ‘Mutant’ button. In the next step, the user needs to enter the sequence position number to generate the single mutant analogs of the peptide sequence at that position. The result is displayed in a tabular format having sequence of the mutant analog its prediction score.

The ‘HemoPI’ mobile app is provided in standard “apk” file format, which can be downloaded from http://crdd.osdd.net/raghava/hemopi/HemoPI.apk. Since this app is currently not available on Google play services, the users should at first allow it to be installed on their android device by going to their security tab in settings and allow third party apps to be installed. The app does not access any sensitive information on the mobile except its own resources.

## Discussion

The aim of this study was to develop an *in silico* prediction method for predicting hemolytic nature of therapeutic peptides. The conversion of potential peptide lead molecules into drug molecules is heavily dependent on their non-hemolytic activity apart from their therapeutic activity. An ideal therapeutic peptide molecule should possess high therapeutic index i.e. high therapeutic activity and minimum hemolytic activity. However, a number of potential therapeutic peptides have been found hemolytic to varied extent. Therefore, over the last decade, much attention has been focused to minimize the hemolytic potency of these peptides without compromising their therapeutic activity. In an experimental lab, this can be achieved by generating different analogs of existing therapeutic peptide followed by screening of these analogs for their low hemolytic activity. The whole procedure is not only time-consuming but labor-intensive also. Therefore, *in silico* tool, which can predict whether a peptide will be hemolytic or not before its synthesis, will not only be economical but also will save the time and labor consumed in their synthesis and evaluation. In this regard, we have made an attempt to develop a dedicated computational method, which can predict whether a given peptide exhibits hemolytic activity or will be non-hemolytic in nature.

**Figure 5:**
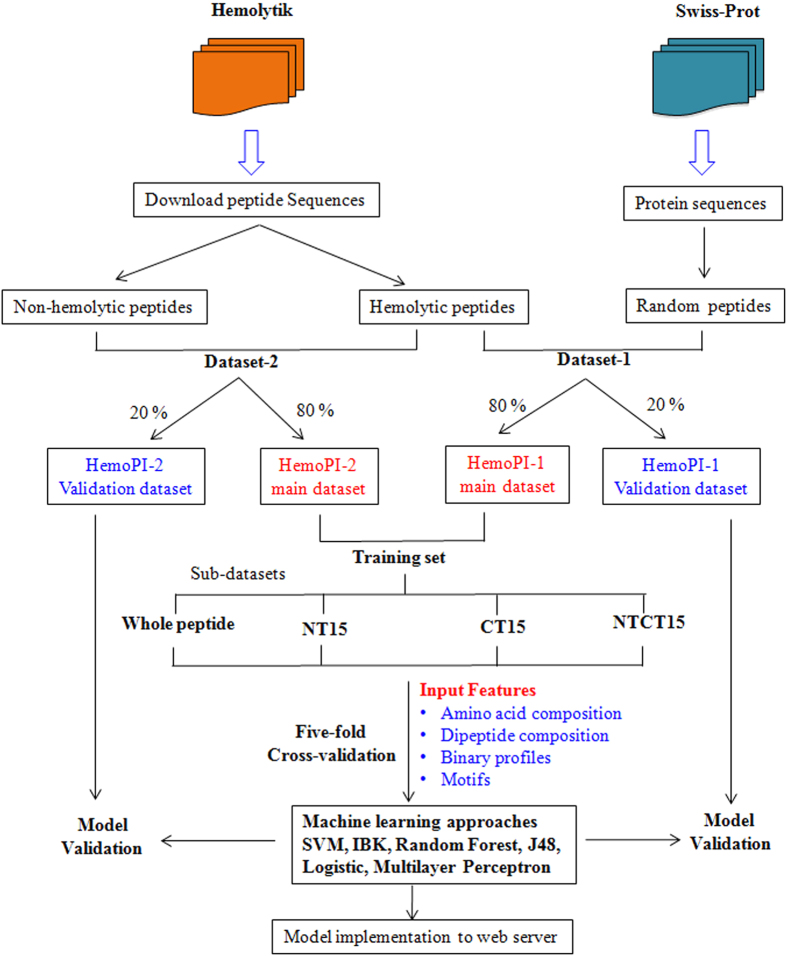
Schematic representation of HemoPI approach.

In order to discriminate hemolytic peptides from non-hemolytic peptides, we have used various machine learning classifiers to develop models as shown in Fig. 5. All machine learning approaches require positive and negative datasets for training. Therefore, dataset generation is very important. In this study, we have downloaded the sequences of hemolytic peptides from Hemolytik database and considered these sequences as positive examples (HemoPI-1). Creating negative dataset is equally important. Since, very few peptides are reported in the literature, which are non-hemolytic; therefore, we generated random sequences from Swiss-Prot proteins of similar length to hemolytic peptides and considered them as negative examples. Though there is a possibility that few of these random sequences may be hemolytic, but the probability is very low. Also this approach of creating negative dataset is used in many previous methods^15^,^19^, especially when negative examples are not available in the literature. Next, to make it more reliable, we have developed another datasets HemoPI-2 and HemoPI-3, where negative dataset is different from that of the HemoPI-1. In these datasets, all the peptide sequences and their hemolytic activity were downloaded from Hemolytik database as well as from DBAASP v.2 database and the sequences were classified into positive and negative datasets. One of the major limitations is that there is no clear definition of hemolytic peptides reported in the literature. Therefore, to overcome this limitation, we have set a few criteria to define hemolytic and non-hemolytic peptides as described in methods. By doing so, all highly hemolytic peptide sequences were considered as positive dataset and rest poor hemolytic and non-hemolytic peptides were considered as negative datasets. The models developed on this dataset will discriminate high hemolytic peptides from poor hemolytic peptides. An overall prediction approach is shown in Fig. 5.

Preliminary sequence analysis using sequence logo revealed few residues (K, L, F, W, G), which are dominant in hemolytic peptides. Most of these residues are generally hydrophobic in nature and are prominent in both N- and C-terminal regions of hemolytic peptides. The importance of hydrophobic residues towards the hemolytic activity of antimicrobial peptides has already been reported previously^20^,^38^,^39^,^40^. In fact, new and potent antimicrobial peptides have been designed by substituting residues that reduce the overall lipophilicity of the peptides^20^,^38^. The differences in the residue preference of hemolytic and non-hemolytic peptides prompted us to develop SVM models using amino acid composition, dipeptide composition and binary profiles as input features. Models developed with these features performed reasonably well. However the performance of all models developed on HemoPI-1 main dataset was better than the models developed on HemoPI-2 main dataset. This is due the fact that in HemoPI-2, positive examples consist of peptides with high hemolytic potencies, while negative examples consist of peptides, which are analogs of hemolytic peptides used as positive examples exhibiting no, or low hemolytic potencies. Therefore, peptides in both the classes contain similar properties including amino acid composition and thus they are difficult to discriminate.

We have also extracted various motifs, which are present in hemolytic (e.g., “ALW”, “GCS”, “AAAK”, etc.) and non-hemolytic (e.g., “KKG” and “QN”) peptide sequences. We have developed a hybrid model by combining the motif information with our machine learning approach to improve the prediction of hemolytic peptides. We have also evaluated the performance of our best models on validation dataset. Our models performed reasonably well on validation dataset suggesting that the models can discriminate hemolytic and non-hemolytic sequences with good accuracy in real life. The implementation of the algorithm in the form of a user-friendly web server allows the experimental researchers to predict and even design a peptide with desirable hemolytic property. HemoPI service is also available in the form of JAVA-based standalone software as well as mobile application, which can be used on the go.

## Conclusion

HemoPI is a unique and much needed *in silico* method, which can predict hemolytic nature of peptides. Optimization of hemolytic properties of peptide lead molecules is very essential but difficult, time-consuming and labor-intensive task in wet laboratory. In this regard, HemoPI will assist the experimental biologists by predicting hemolytic properties of peptides before their synthesis. HemoPI will also be useful in designing peptides or their analogs with desired non-hemolytic activity. Therefore, we believe that development of HemoPI will accelerate the peptide-based drug discovery.

## Limitations and Future Prospects

In the present study, we have made an attempt to develop a much needed *in silico* method to predict hemolytic nature of the peptides. Development of datasets of hemolytic and non-hemolytic peptides is very challenging. One of the limitations is that there is no clear definition of hemolytic peptides in the literature. Most of the peptides (except few) show hemolysis to some extent at some concentration (may be at higher concentration). From the collected data, it was found that same peptide was reported to cause hemolysis to different extents in different studies. Another limitation is that peptides have been tested at different concentrations in different studies with different units like EC_50_, HC_50_, MHC and % hemolysis, etc, which make it more difficult to decide which peptide is hemolytic and which is non-hemolytic. It is also observed that a peptide shows 10% hemolysis at 50 μM concentration while another shows 9% hemolysis at 10 μM. Therefore, it is not easy to decide which one is hemolytic and which one is non-hemolytic? In addition, there is no uniformity in the data reported in the literature. Ideally, hemolysis should be evaluated at different concentrations of a peptide and its HC_50_ should be calculated but in most of the cases only a single concentration has been reported in research articles. So it becomes even more challenging to classify the peptides as hemolytic or non-hemolytic.

Though we have used the latest data for developing models, our team will update the datasets as soon as the new data is available. In addition, we will compare our method with new methods, if any developed in the future.

## Additional Information

**How to cite this article**: Chaudhary, K. *et al*. A Web Server and Mobile App for Computing Hemolytic Potency of Peptides. *Sci. Rep.* **6**, 22843; doi: 10.1038/srep22843 (2016).

## Supplementary Material

Supplementary Information

## Figures and Tables

**Table 1:** The performance of amino acid composition-based models developed using various machine learning techniques for predicting hemotoxicity of peptides.

Methods	Sn (%)	Sp (%)	Acc (%)	MCC
HemoPI-1 main dataset				
SVM	95.7	94.8	95.3	0.91
IBK	95.5	93.7	94.6	0.89
Multilayer Perceptron	93.9	92.8	93.3	0.87
Logistic	93.4	93.7	93.6	0.87
J48	89.6	88.5	89.0	0.78
Random Forest	94.1	94.6	94.3	0.89
HemoPI-2 main dataset				
SVM	76.0	76.8	76.4	0.53
IBK	75.6	76.0	75.7	0.51
Multilayer Perceptron	74.0	74.1	74.0	0.48
Logistic	64.5	68.1	66.1	0.32
J48	79.0	60.0	70.3	0.40
Random Forest	77.8	77.8	77.8	0.56

These models were developed and evaluated using five-fold cross-validation.

**Sn**: Sensitivity; **Sp**: Specificity; **Acc**: Accuracy; **MCC**: Matthews correlation coefficient.

**Table 2:** Performance of amino acid composition-based SVM models for predicting hemotoxicity of peptides using five-fold cross-validation.

Approach	Sn (%)	Sp (%)	Acc (%)	MCC
HemoPI-1 main dataset				
NT10	93.8	92.9	93.3	0.86
CT10	84.4	89.0	87.0	0.74
NTCT10	95.7	94.0	94.9	0.90
NT15	93.8	95.7	94.8	0.90
CT15	89.7	93.8	91.8	0.84
NTCT15	96.5	96.1	96.3	0.93
HemoPI-2 main dataset				
NT10	71.6	71.1	71.4	0.43
CT10	67.7	70.1	68.8	0.38
NTCT10	77.9	77.0	77.5	0.55
NT15	74.9	73.9	74.5	0.49
CT15	74.3	69.9	72.4	0.44
NTCT15	77.7	76.2	77.0	0.54

**Sn**: Sensitivity; **Sp**: Specificity; **Acc**: Accuracy; **MCC**: Matthews correlation coefficient; **NT10**: first 10 residues from N-terminus; **CT10**: last 10 residues from C-terminus; **NTCT10**: NT10+CT10.

**Table 3:** Performance of dipeptide composition-based SVM models for predicting hemotoxicity of peptides using five-fold cross-validation.

Approach	Sn (%)	Sp (%)	Acc (%)	MCC
HemoPI-1 main dataset				
Whole peptide	94.6	94.3	94.5	0.89
NT10	87.0	96.8	92.5	0.85
CT10	87.5	90.1	89.0	0.78
NTCT10	94.1	94.0	94.0	0.88
NT15	92.7	94.7	93.8	0.88
CT15	90.4	92.8	91.6	0.83
NTCT15	94.8	95.1	95.0	0.90
HemoPI-2 main dataset				
Whole peptide	77.6	79.2	78.3	0.57
NT10	69.8	69.7	69.8	0.39
CT10	69.1	69.8	69.4	0.39
NTCT10	73.2	81.7	77.0	0.55
NT15	75.3	72.5	74.1	0.48
CT15	71.2	78.3	74.3	0.49
NTCT15	78.6	79.5	79.0	0.58

**Sn**: Sensitivity; **Sp**: Specificity; **Acc**: Accuracy; **MCC**: Matthews correlation coefficient; **NT10**: first 10 residues from N-terminus; **CT10**: last 10 residues from C-terminus; **NTCT10**: NT10+CT10.

**Table 4:** Performance of binary profile-based SVM models for predicting hemotoxicity of peptides using five-fold cross-validation.

Approach	Sn (%)	Sp (%)	Acc (%)	MCC
HemoPI-1 main dataset				
NT10	90.5	96.1	93.7	0.87
CT10	85.0	93.4	89.6	0.79
NTCT10	93.8	97.5	95.7	0.91
NT15	92.7	97.0	95.0	0.90
CT15	89.0	91.5	90.3	0.81
NTCT15	94.0	96.7	95.3	0.91
HemoPI-2 main dataset				
NT10	70.7	72.2	71.4	0.43
CT10	70.0	71.4	70.6	0.41
NTCT10	78.9	74.6	77.1	0.54
NT15	70.9	75.8	73.1	0.46
CT15	67.5	77.4	71.8	0.45
NTCT15	77.0	77.0	77.0	0.54

**Sn**: Sensitivity; **Sp**: Specificity; **Acc**: Accuracy; **MCC**: Matthews correlation coefficient; **NT10**: first 10 residues from N-terminus; **CT10**: last 10 residues from C-terminus; **NTCT10**: NT10+CT10.

**Table 5:** Performance of hybrid model for predicting hemotoxicity of peptides.

Datasets	Sn (%)	Sp (%)	Acc (%)	MCC
HemoPI-1 main	96.0	94.6	95.3	0.91
HemoPI-2 main	78.3	77.6	78.0	0.56
HemoPI-1 validation	96.4	99.1	96.4	0.93
HemoPI-2 validation	78.2	78.3	75.7	0.51

**Sn**: Sensitivity; **Sp**: Specificity; **Acc**: Accuracy; **MCC**: Matthews correlation coefficient.
