# Supplementary material for: A Web Server and Mobile App for Computing Hemolytic Potency of Peptides
Source: Sci Rep. 2016 Mar 8;6:22843. doi: 10.1038/srep22843 (PMC4782144; doi:10.1038/srep22843)
Supplement: Supplementary Information [file srep22843-s1.pdf]

A Web Server and Mobile App for Computing Hemolytic Potency of Peptides

Kumardeep Chaudhary[#,1], Ritesh Kumar[#,2], Sandeep Singh[#,1], Abhishek Tuknait[#,1], Ankur Gautam[1], Deepika Mathur[1], Priya Anand[1], Grish C. Varshney[3] and Gajendra P.S. Raghava[*,1]

[1]Bioinformatics Centre, CSIR-Institute of Microbial Technology, Sector 39A, Chandigarh, India

[2]CSIR-Central Scientific Instruments Organisation, Sector 30C, Chandigarh, India

[3]Cell Biology and Immunology Division, CSIR-Institute of Microbial Technology, Chandigarh, India

# Authors contributed equally

Supplementary Information

Figure S1. Comparison of amino acid composition between HemoPI-1 dataset, random negative datasets (Random1-5) dataset, and Swiss-Prot.

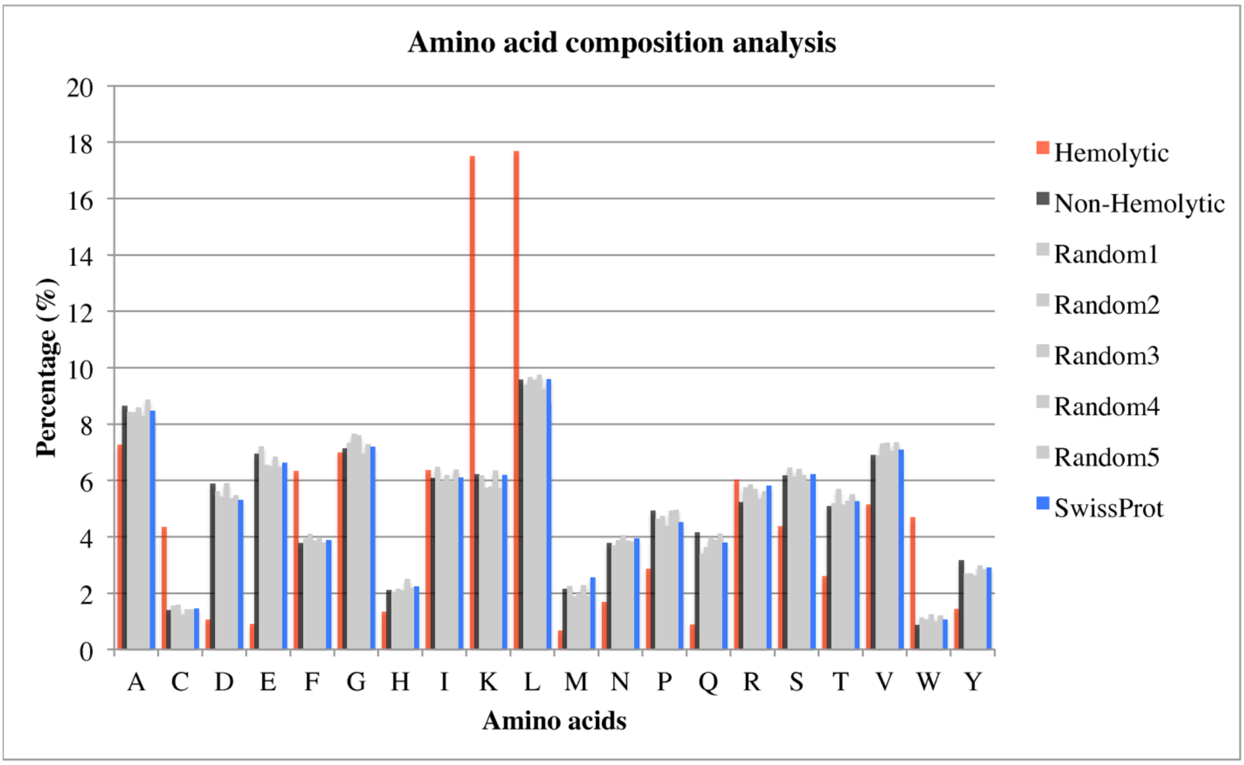

Table S1: Rules for highly and poorly hemolytic peptides for HemoPI-3.

Highly Hemolytic Peptides		Poorly Hemolytic Peptides	
**Hemolysis**	**Concentration**	**Hemolysis**	**Concentration**
≥ 5%	≤10µM	≤ 2%	≥ 10µM
≥ 10%	≤ 20µM	≤ 5%	≥ 20µM
≥ 15%	≤ 50µM	≤ 10%	≥ 50µM
≥ 20%	≤ 100µM	≤ 15%	≥ 100µM
≥ 30%	≤ 200µM	≤ 20%	≥ 200 µM
≥ 50%	≤ 300µM	≤ 30%	≥ 300µM
MHC	≤ 50µM	≤ 50%	≥ 500µM
		MHC	≥ 100µM

Table S2. Performance of amino acid composition-based SVM model using five-fold cross-validation on HemoPI-3 main dataset

Approach	Sn%	Sp%	Acc%	MCC
**Whole peptide**	79.24	76.48	77.98	0.56
**NT10**	70.33	66.74	68.71	0.37
**CT10**	70.38	65.42	68.12	0.36
**NTCT10**	77.83	74.91	76.52	0.53
**NT15**	75.71	71.52	74.05	0.47
**CT15**	71.37	73.98	72.42	0.45
**NTCT15**	82.81	78.23	81.00	0.61

Performance of amino acid composition based model on validation dataset.

Approach	Sn%	Sp%	Acc%	MCC
**Whole peptide**	81.92	71.43	77.16	0.54
**NTCT15**	79.72	64.52	73.73	0.45

Sn: Sensitivity; **Sp**: Specificity; **Acc**: Accuracy; **MCC**: Matthews correlation coefficient.

Table S3. Performance of whole peptide amino acid composition based models using different machine learning algorithms on HemoPI-3 dataset.

Method	Sn%	Sp%	Acc%	MCC
**SVM**	79.24	76.48	77.98	0.56
**IBK**	77.54	71.24	74.67	0.49
**Multilayer Perceptron**	71.19	73.10	72.06	0.44
**Logistic**	66.81	64.97	65.97	0.32
**J48**	72.88	67.17	70.28	0.40
**Random Forest**	80.37	77.16	78.91	0.57

Sn: Sensitivity; **Sp**: Specificity; **Acc**: Accuracy; **MCC**: Matthews correlation coefficient.

Table S4. Performance of amino acid composition-based SVM models for Random1-5 main datasets for predicting hemotoxicity of peptides using five-fold cross-validation.

Datasets	Sn (%)	Sp (%)	Acc (%)	MCC
HemoPI-1 Random1	91.4	96.6	94	0.88
HemoPI-1 Random2	94.8	94.8	94.8	0.90
HemoPI-1 Random3	94.3	93.2	93.8	0.88
HemoPI-1 Random4	93	94.6	93.8	0.88
HemoPI-1 Random5	95.3	94.6	95	0.90

Sn: Sensitivity; **Sp**: Specificity; **Acc**: Accuracy; **MCC**: Matthews correlation coefficient.

Table S5. Performance of dipeptide composition-based SVM models using five-fold cross-validation on HemoPI-3 main dataset

Approach	Sn%	Sp%	Acc%	MCC
**Whole peptide**	78.39	78.68	78.52	0.57
**NT10**	71.98	68.74	70.51	0.41
**CT10**	72.47	67.08	70.01	0.40
**NTCT10**	79.76	77.12	78.58	0.57
**NT15**	74.29	76.16	75.03	0.50
**CT15**	72.94	75.44	73.94	0.48
**NTCT15**	81.93	83.06	82.38	0.64

Performance of dipeptide composition based model on validation dataset

Approach	Sn%	Sp%	Acc%	MCC
**Whole peptide**	77.97	70.07	74.38	0.48
**NTCT15**	76.22	67.74	72.88	0.44

Sn: Sensitivity; **Sp**: Specificity; **Acc**: Accuracy; **MCC**: Matthews correlation coefficient.

Table S6. Performance of binary profile-based SVM models using five-fold cross-validation on HemoPI-3 main dataset

Approach	Sn%	Sp%	Acc%	MCC
**NT10**	71.61	68.29	70.11	0.40
**CT10**	68.47	69.36	68.87	0.38
**NTCT10**	80.80	75.09	78.25	0.56
**NT15**	74.70	73.68	74.30	0.48
**CT15**	75.29	70.76	73.47	0.46
**NTCT15**	83.16	76.61	80.57	0.60

Performance of binary composition based model on validation dataset.

Approach	Sn%	Sp%	Acc%	MCC
**NTCT15**	81.82	64.52	75.00	0.47

Sn: Sensitivity; **Sp**: Specificity; **Acc**: Accuracy; **MCC**: Matthews correlation coefficient.
